# Extracellular Vesicles as Conduits of Non-Coding RNA Emission and Intercellular Transfer in Brain Tumors

**DOI:** 10.3390/ncrna5010001

**Published:** 2018-12-25

**Authors:** Cristiana Spinelli, Lata Adnani, Dongsic Choi, Janusz Rak

**Affiliations:** The Research Institute of the McGill University Health Centre, Montreal, QC H4A 3J1, Canada; cristiana.spinelli@mail.mcgill.ca (C.S.); lata.adnani@gmail.com (L.A.); choi.dongsic@mail.mcgill.ca (D.C.)

**Keywords:** non-coding RNA, microRNA, RNA biotypes, extracellular vesicles, exosomes, brain tumors, glioblastoma, cell communication, biomarkers, oncogenes

## Abstract

Non-coding RNA (ncRNA) species have emerged in as molecular fingerprints and regulators of brain tumor pathogenesis and progression. While changes in ncRNA levels have been traditionally regarded as cell intrinsic there is mounting evidence for their extracellular and paracrine function. One of the key mechanisms that enables ncRNA to exit from cells is their selective packaging into extracellular vesicles (EVs), and trafficking in the extracellular space and biofluids. Vesicular export processes reduce intracellular levels of specific ncRNA in EV donor cells while creating a pool of EV-associated ncRNA in the extracellular space and biofluids that enables their uptake by other recipient cells; both aspects have functional consequences. Cancer cells produce several EV subtypes (exosomes, ectosomes), which differ in their ncRNA composition, properties and function. Several RNA biotypes have been identified in the cargo of brain tumor EVs, of which microRNAs are the most studied, but other species (snRNA, YRNA, tRNA, and lncRNA) are often more abundant. Of particular interest is the link between transforming oncogenes and the biogenesis, cargo, uptake and function of tumor-derived EV, including EV content of oncogenic RNA. The ncRNA repertoire of EVs isolated from cerebrospinal fluid and serum is being developed as a liquid biopsy platform in brain tumors.

## 1. Pathways of Intercellular Communication in Brain Tumors

The recent World Health Organization (WHO) 2016 classification of brain tumors identifies over 155 distinct disease entities grouped into 16 different classes [1]. While primary brain tumors dominate this spectrum with notable age-related variability in nature and incidence, metastatic brain tumors are both highly prevalent and deadly [2,3,4]. While progress has been achieved in treatment of several brain malignancies, including advances in surgical intervention, radiation, chemotherapy and targeted agents, such gains have been minimal in high grade brain tumors with infiltrative growth patterns, such as glioblastoma (GBM), where prognosis remains grim in spite of intense research efforts [2]. Similarly, in high-grade pediatric gliomas, several subgroups of medulloblastoma, and in primitive embryonal brain tumors therapeutic challenges, do exist and remain largely unmet [5,6,7]. 

Arguably the intractable nature of some of these disease states may be, at least in part, attributable to a particularly complex nexus of biological mechanisms involved, with formation of distinct, heterogeneous and evolving multicellular ecosystems endowed with the ability to adapt to therapy and evade eradication [2,8,9]. One aspect of this complexity lies in the fact that high-grade brain tumors are comprised of intrinsically heterogeneous hierarchies of transformed cell population including brain tumor initiating cells (BTICs), their progeny [10,11], adjacent resident cells of the brain neuropile (astrocytes, microglia), stromal cells (endothelial cells and pericytes) and transitory cellular populations recruited from circulating blood and the immune system (inflammatory and immune cells) [12]. In certain cases, such as GBM, the contribution of host cells is estimated to be as high as 30–40% of the total tumor mass [13], with untold phenotypic diversity and functional intricacies of resulting cellular communication and signalling networks [14,15]. 

Intercellular communication in brain malignancies is far from being systematically mapped. However, several core plausible mechanisms have been revealed over the years including the canonical (‘molecular’) and more complex (‘structural’) interactive networks. The former is exemplified by multiple ligand-receptor or effector-substrate systems responsible for molecularly precise, but limited in scope interactions of autocrine, juxtacrine, paracrine and endocrine nature. These mechanisms regulate cellular behaviour, such as proliferation and self-renewal of BITCs, differentiation of their progeny, cell survival, stress pathways, migration and invasion, as well as mounting stromal, inflammatory and vascular responses [16]. 

Examples of molecular networking in brain tumors have been extensively reviewed in the recent literature [12] and deserve but a handful of updates. For instance, in one study autocrine release of the CCL5 chemokine regulated by Neurofibromatosis type I (NF1) gene has been implicated in sustaining mesenchymal GBM cell survival [17]. Another class of guidance molecules exemplified by Semaphorin 3A (Sema3A) was found to regulate GBM cell migration [18]. Autocrine, juxtacrine and paracrine interactions in GBM also involve other chemokines (CXCL12), cytokines (EGF, TGFa, PDGF, HGF/SF), adhesion molecules and other factors [12]. Among them some of the canonical signalling networks encompassing BMP/TGFβ, Wnt, Notch-Delta, JAK-STAT and sonic hedgehog (SHH) pathways may represent relics of developmental regulation, or deregulated pathways of tissue homeostasis activated during brain tumorigenesis, along with circuits that regulate myeloid cell recruitment, immunoregulation (CSF1), neuroinflammation and angiogenesis (VEGF, angiopoietins) among other effects [12,19]. The intercellular cooperation among GBM cells has also been highlighted by the effects of leukemia inhibitory factor (LIF) and IL-6 on the maintenance of the balance between tumor cells expressing oncogenic mutation of the epidermal growth factor receptor variant III (EGFRvIII) and those with the wild type *EGFR* gene [20]. In another recent study a reciprocal paracrine interaction between glioma stem cells (GSCs) and their progeny was attributed to activities of factors, such as neurosecretory protein, VGF, and brain-derived neurotrophic factor (BDNF) [21], each case involving a specific ligand-receptor pathway, as well as down-stream programs they activate. 

‘Structural’ forms of intercellular communication have emerged as a form of multimolecular (rather than unicellular) exchange of information between cells. In this case specialized physical cell-to-cell interfaces serve as gateways to transfer multiple molecular components between cells, including through junctions [22], tunneling nanotubes (TNTs) [8,23], or tumor microtubes (TMs) [24]. Molecular mechanisms involved in formation of these membrane structures in not always well understood, but some of the respective regulators include connexins (CX43) [22], actin, myosin, GAP43, TTHY1 and other molecules with various cellular roles [8]. Unlike growth factor networks, structural interactions permit cellular exchange of a much wider spectrum of bioactive molecules, ranging from ions and small molecular mediators to integral cellular or membrane proteins, nucleic acids and organelles, such as intracellular vesicles, mitochondria and nuclei [24,25]. While these direct cell–cell contacts enable intercellular communication at the local or microregional level [8], another form of large-scale molecular transfer has evolved to connect cells over both short and long distances through release and uptake of membrane structures known as extracellular vesicles (EVs) [13,16]. Since EVs represent a unique conduit for intercellular transmission of nucleic acids, including non-coding RNA, their related properties will be the focus of our remaining comments.

## 2. Extracellular Vesicles (EVs) as Molecular Information Carriers

All cells have the capacity to release multimolecular structures generally referred to as extracellular particles (EPs). Of those a unique and large segment consists of EVs, defined as spherical or elongated vesicular structures with luminal center surrounded by the plasma membrane bilayer [26]. These features are consistent with subcellular sites of origin attributed to several EV subpopulations described thus far [27], such as plasma membrane on the cell surface and intracellular vesicular systems, especially the endosome [26,28]. 

**Figure 1:**
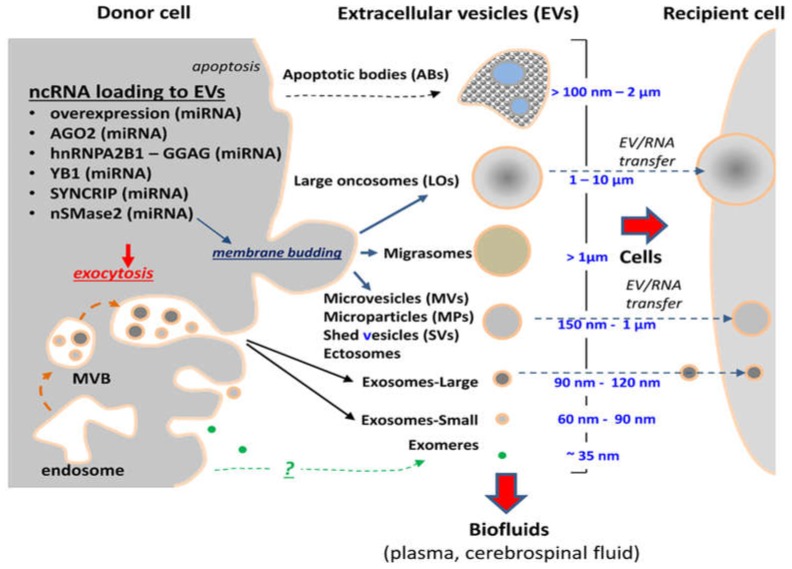
Heterogeneity of extracellular vesicles as carriers of non-coding RNA. The repertoire of EVs produced by cancer cells including different subsets of brain tumors and their stem cell populations creates a platform for multiple mechanisms of non-coding RNA release. Some of the reported pathways are listed and described in the text. The ncRNA biotypes linked to indicated mechanisms of EV packaging are given in parentheses. However, knowledge of such packaging processes beyond microRNA is presently very limited. EVs serve as vehicles to eject cellular content and/or transmit their RNA cargo between donor and recipient cells.

EVs are highly heterogeneous in size, molecular content, biogenetic origin, properties and biological activity (Figure 1). The size range for most EVs found in cellular supernatants falls between 30 nm and over 1000 nm. Larger EVs have also been described, as exemplified by large oncosomes (LOs), EVs measuring several microns in diameter and associated with ameboid cell migration of certain types of cancer cells including glioma [11]. Traditionally, three major classes of EVs have been described in the literature as key components of the vesicular secretome of various cellular populations [29] and distinguished according to their physical characteristics, biogenetic mechanisms and some antigenic or molecular markers [11,26,30]. In this regard, the outward budding of vesicular structures from the plasma membrane is regarded as a source of EVs referred to as ectosomes or microvesicles (MVs) that range in size between 150–1000 nm and are often molecularly reminiscent to their parental cell [13]. 

In contrast, the expulsion of small vesicles generated within segments of the cellular endosome known as multivesicular bodies (MVBs) is believed to give rise to smaller EVs (30–150 nm in diameter) often referred to as exosomes [26,28,30]. Exosomes possess distinct physical properties, as revealed by their ability to sediment at high centrifugal forces of 1 × 10^5^× *g*, or greater, and to float at 1.11–1.19 g/mL density in sucrose and iodixanol gradients [31]. They also exhibit a specific set of molecular markers dominated by membrane tetraspanins (CD81, CD82, CD63 and CD9), and proteins associated with the endosomal sorting complexes required for transport (ESCRT) system (TSG101, ALIX), as well as other features identified in their proteome [30]. Some of these properties may be shared between recently described fractions of large and small exosomes [32] and with other exosome-like small EVs produced by cancer and normal cells [30,33]. As in the case of other EVs, exosomes contain complex molecular cargo including proteins, lipids, carbohydrates, and nucleic acids (RNA and DNA), but their composition is often different than that of parental cells [30] and, thereby, a subject of distinct regulatory influences [26]. 

The diverse phenotypes of exosomes and ectosomes are not accidental, and likely reflect only partially mapped pathways involved in their biogenesis [26]. It is generally accepted that ectosomes are generated at the plasma membrane through lipid asymmetry and curvature modulating mechanisms. Some of the molecular effectors implicated in these processes include: ARF6 GTPase, phospholipase D (PLD), myosin light chain kinase (MLCK), RHOA, LIMK1, lipid scramblase (TMEM16F), acidic sphingomyelinase, Rab22, arrestin domain-containing protein 1 (ARRDC1) and other proteins whose manipulation alters MV output [34,35,36,37,38,39,40]. It has also been proposed that budding events may be triggered by physical consequences of protein accumulation at the cellular plasma membrane [41]. 

On the other hand, exosome biogenesis forms several increasingly well mapped pathways [26,28]. Those include ESCRT-dependent and ESCRT-independent intracellular mechanisms, some of which may depend on neutral sphingomyelinase activity and/or on expression of tertraspanins in EV-generating endosomal membranes. Intracellular trafficking of MVBs containing exosome precursor EVs (known as intraluminal vesicles—ILVs) and their fusion with the plasma membrane results in exosome release, a process involving several documented molecular players, such as Rab35/Rab1, Rab27a/b and Rab7, as well as SNAREs and other effectors [26,42]. There is also emerging evidence as to autophagy-related proteins, such as ATG12, in the control of the vesiculation process [43].

Apoptotic bodies (ABs) comprise a distinct subtype of EVs found in the vesicular secretome and biofluids that have been in contact with dying cells. Indeed, ABs represent remnants of cells that have undergone programmed death, which fundamentally separates them from other EVs that are mostly produced by viable cellular populations. 

While distinction of various aforementioned EV categories may help catalogue their most obvious properties, the true depth of EV heterogeneity is likely far greater, as revealed by improved methods of EV fractionation [30,32], high-resolution flow cytometry and EV immunophenotyping [44,45]. Also, the intrinsic complexity of the EV proteome predicts the existence of dozens of (rather than three) EV subsets [46,47,48,49]. This diversity could be further enlarged, considering the heterogeneity of nucleic acid (RNA or DNA) distribution among EV subpopulations [50,51,52,53] (as discussed below). 

EVs play complex biological roles in intra-cellular and inter-cellular homeostasis (Figure 1). The former is exemplified by the ability of the EV biogenesis pathways to bypass lysosomal degradation and mediate the expulsion of the molecular surplus from their parental cells into their exterior, thereby relieving the associated synthetic, metabolic, or signalling stresses [54,55,56,57]. These cell-autonomous effects are intertwined with their intercellular consequences. Thus, exported EVs naturally come into contact with multiple cells and structures in the local microenvironment and systemically, and these interactions may either elicit biological effects or lead to EV degradation [13,16,58,59]. 

There are several ways in which EVs may affect the homeostasis of cells with which they interact. For example, they may release their bioactive content near target cells resulting in a burst of related effects dependent on the respective cellular receptors and substrates. Intact EVs may also make surface contact with the target cell, thereby engaging the corresponding membrane-associated receptors [16,60,61]. EVs can also fuse with the plasma membranes of recipient cells and transfer some of their molecular repertoire to their surfaces and cytoplasms resulting in the transfer of the corresponding functions [62]. Migrating cells may deposit their EVs along extracellular membrane and matrix tracks, which leads to recognition of such vesicular ‘footprints’ and responses to them of cells that migrate in the same direction [63,64]. The most studied, however, are several pathways that enable EV internalization and trafficking within the recipient cells, and into different intracellular compartments, such as endosome, lysosome, cytosol or the nucleus [60,65]. The exact regulation of these events is unclear and may be dictated by characteristics of both EVs and recipient cells. For example, recent studies suggested a role of specific interactions between EVs and cellular membrane receptors (e.g., proteoglycans) followed by EV engulfment through phagocytosis, endocytosis or macropinocytosis [65]. Some of the regulators of these events include RAS, ERK and PI3K pathways [46,66,67]. The intake of molecular content encapsulated within EVs may result in reprogramming of recipient cells with changes in their signalling patterns and phenotype due to re-utilization of EV-associated functional proteins or nucleic acids. However, EV uptake may also elicit stress reposes or cargo degradation in the lysosome [65].

## 3. The Emerging Role of Extracellular Vesicles as Biological Regulators in Brain Tumor Progression

Processes of EV-mediated intercellular communication are profoundly altered in brain tumors, with GBM serving as one of the earliest paradigms to be identified and among the most studied [68]. Indeed, oncogenic transformation [45,68], epigenetic reprogramming, aberrant differentiation [69] and microenvironmental stresses [70] influence all aspects of cellular vesiculation, including EV release, immunophenotype, proteome, RNA and DNA content, as well as their uptake by recipient cells and function [46]. These changes contribute to the increasingly appreciated involvement of EVs in various aspects of biological regulation and cancer progression, including both primary and metastatic brain tumors [71,72,73]. These functional responses are mediated by various components of the EV cargo (likely simultaneously) such as proteins, nucleic acids and/or lipids [13]. Examples of such EV-associated multimolecular effects include growth regulation in the case of glioma cells and stem cells [68,74], angiogenesis [52,69], neuroinflammation [35,75,76], brain metastasis [58,72], drug resistance [9], deregulation of immune responses [77] and several other important processes [13]. 

Uniquely, cancer cell-derived EVs contain mutant, oncogenic or otherwise disease-specific and pathological proteins and nucleic acids [16]. Intercellular transfer of this material may result in states resembling horizontal transformation or induce profound changes in the phenotype of recipient cells, either transformed or non-transformed [66,68]. For example, EVs derived from glioma cells carry oncogenic EGFRvIII in a form of protein, RNA and DNA, all of which may be transferred to recipient cells [33,52,68,78,79,80]. Glioma EVs may also contain wild-type EGFR [81], PTEN [82], and mutant IDH1 [83], while EVs of medulloblastoma cells carry DNA comprising MYC amplicon (as reviewed in [46]). In some of these studies uptake of oncogenes or tumor suppressors via EV transmission resulted in deregulation of signalling pathways, gene expression and genetic instability [68,82,84,85]. 

In addition to functional effects resulting from EV-mediated intercellular transfer of oncoproteins, also mutant DNA and oncogenic coding and non-coding RNA sequences undergo intercellular trafficking. These elements of the EV cargo have attracted considerable attention as sources of brain tumor-associated biological abnormalities, as well as their biomarkers [52,68,86,87,88]. 

RNA constitutes an important part of both functional and biomarker aspects of brain tumor-derived EVs. They are being explored as a part of a larger effort to understand and harness the intercellular regulatory milieu afforded by extracellular RNA in the brain tumor context [89]. The presence of RNA in the extracellular space was initially described by Kolodny (1971) who suggested, then unspecified, mechanisms of stabilization and protection of this material from ribonucleases (RNAses) [90]. EVs and RNA binding proteins (RBPs) represent two currently appreciated forms of such a protection. Subsequently, several studies discovered exRNAs in bodily fluids such as blood, saliva, breast milk and urine, thereby establishing the remarkable commonality of such a release process [91,92,93,94,95]. We will restrict our remaining comments to EV-mediated extracellular release of RNA in view of the uniqueness of this mechanism and the emerging roles of EVs brain tumors [96].

## 4. EV-Associated RNA Biotypes

**Figure 2:**
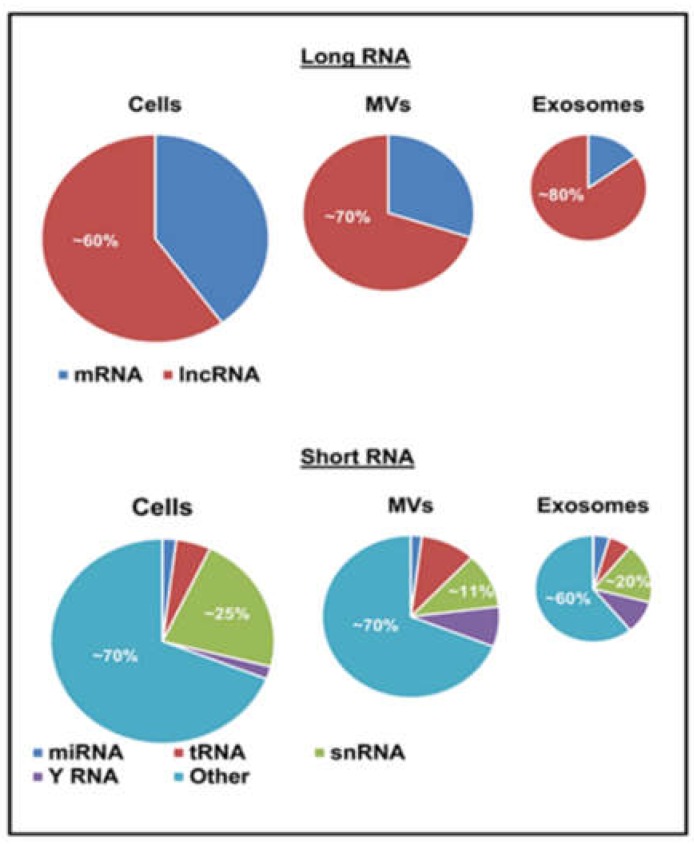
Approximate distribution of RNA biotypes in glioma cells and their derived extracellular vesicles. The proportions of different RNA biotypes differ between cells, microvesicles (MVs) and exosomes (see text-adapted from [100,101]).

EV preparations contain distinctive repertoires of RNA species, including transcripts and non-coding RNA [50,51,52,97]. The latter category is highly diverse and includes long non-coding RNA (lncRNA) as well as several small RNA biotypes (≤200 nucleotides), such as microRNAs (miRNAs), piwi protein interacting RNA (piRNA), small nuclear RNA (snRNA), small nucleolar RNA (snoRNA), small Cajal body-specific RNA (scaRNA), circular RNA (circRNA), Y RNA, natural antisense RNA (asRNA), ribosomal RNA (rRNA), and vault RNA (vRNA) [13,98,99]. Notably, while cellular RNA profiles are enriched in mRNA (Figure 2) with a preponderance of full-length transcripts of different lengths, EVs contain mostly shorter RNA species (≤200 nucleotides) including short transcripts, fragmented mRNA and small RNA biotypes (miRNA, snoRNA, snRNA, Y RNA, vault RNA, lncRNA and miscellaneous). Since sequencing studies often rely on separate library preparations for long and short RNA the direct comparisons between them are not always straightforward. Nonetheless, these analyses clearly indicate differential profiles of ncRNA subsets between parental cells and EV subpopulations [99,100].

The composition of EV-associated RNA is defined by several key factors. They may include the overexpression of a particular RNA in the parental cell population, the specifics of the biogenetic mechanism involved in formation of a particular RNA-carrying EV subtype and the related engagement of distinct RNA packaging processes, which may be biotype- and/or sequence-specific, or unspecific [13]. Indeed, the repertoire of RNA found in larger budding-type EVs, such as microvesicles and large oncosomes tends to correspond more closely to that of parental cells, while exosomes often cluster apart from these patterns [101]. While the exact mechanisms of RNA loading into EVs still remain poorly characterized, the proposed inclusion of mRNA into the EV cargo is attributed to several determinants, such as overall abundance, fragmentation (at 3’UTR) and the presence of the 3’UTR-associated 25 nucleotide ‘zipcode’, in which a CTGCC core domain on a stem-loop structure binds miR-1289 and thereby directs the transcript to the vesiculation pathway [102].

EV packaging of microRNA is also frequently regulated by the abundance of both the specific microRNA in question and of its mRNA target(s), which may act as cellular microRNA retention signals. Moreover, RNA binding proteins, such as AGO2 [103] and the elements of the RISC-loading (silencing) complex [104] have been reported to contribute to the relative enrichment of certain microRNA in the EV cargo. However, alterative mechanisms have also been proposed [105]. For example, the sumoylated ribonucleoprotein hnRNPA2B1, which recognizes the GGAG motif in a subset of microRNA, was implicated as a mechanism of their exosomal export [106]. In another recent study, Schekman’s group implicated packaging of specific microRNAs (miR-223) into exosome-like EVs positive for CD63 tetraspanin and through a mechanism involving Y-box protein 1 (YB1) [107]. It is of interest to note that YB1 is among the most upregulated genes in pediatric high-grade glioma, along with another vesicle transport-associated protein SNX3 [108], which may suggest that EV-mediated trafficking of regulatory RNAs could play a role in the pathogenesis of this disease, otherwise already linked to profound deregulation of epigenetic circuitry [109]. 

The export of microRNA as EV cargo has also been linked to the activity of synaptotagmin-binding cytoplasmic RNA-interacting protein (SYNCRIP/hnRNP-Q/NSAP1), which directly interacts with a specific motif (hEXO) mandating enrichment of microRNA in the exosomal compartment [110]. Another reported export pathway involves 3’ end uridylation of microRNA, which directs these nucleic acids to sites of EV biogenesis, while adenylation appears to have the opposite effect [111]. Furthermore, overexpression of neutral sphingomyelinase (nSMase2) may increase the extracellular pool of microRNA [112], which may be related to the impact of this enzyme on core mechanisms of exosome biogenesis [113]. It should be noted that a significant proportion of microRNA found in the extracellular space may exist in a form of protein complexes rather than EVs, and that microRNA constitutes a minor fraction of the global RNA content in the EV cargo (Figure 2) [53,100]. Indeed, little is known about the mechanisms of EV-mediated release of other RNA biotypes, including those more abundant (snoRNA, snRNA, lncRNA, Y RNA) [100]. 

## 5. Evidence for the Functional Role of EV-Associated RNA in Cancer

The repertoire of EV-associated RNA reflects the status of parental cells and may serve to communicate these changes to recipient cells [13,16]. For example, the composition of EV-associated transcripts has been linked to factors such as oncogenic transformation, differentiation status, hypoxia, oxidative stress or therapy [70,100,114,115]. Under these various conditions EVs may often become enriched in mRNA species involved in cell viability, immune responses, migration or angiogenesis suggesting the possible role of these EVs in the respective processes [114,116,117,118]. 

While a large proportion of EV-associated mRNA is believed to be fragmented and endowed with mostly regulatory roles [99], there is also evidence in favor of translation and expression of the corresponding proteins in EV recipient cells [13,119]. This has been demonstrated in the case of reporter genes such as luciferase [52], cre recombinase [120] or green fluorescent protein (GFP) [119], as well as for some endogenous transcripts [50,51,121]. An especially elegant demonstration of the EV-mediated mRNA transfer between cells and the subsequent translation was described recently by Lai and colleagues [119]. These authors used a multiplex reporter system based on the simultaneous fluorescent tagging of EV membranes and an independent mRNA tracking system that employed RNA binding viral MS2CP protein and GFP mRNA. With these tools these investigators demonstrated that the EV uptake, mRNA transfer and translation in recipient Gli36 glioma cells occurs within 1-h of EV exposure. Interestingly, in an earlier study EVs derived from embryonic stem cells (ES) were found to be enriched for mRNA encoding several pluripotent transcription factors, which were transferred to recipient hematopoietic cells resulting in changes of cellular phenotype [50]. Other studies have shown that EVs from human colorectal cancer cells (SW480) are enriched in cell cycle-related mRNAs and their intercellular transfer promotes proliferation of endothelial recipient cells. These authors suggested that EV release by cancer cells may be involved in tumor growth and metastasis by facilitating angiogenesis [121].

Functional effects have been reported also in the case of EV-associated microRNA. This is of interest as microRNAs regulate the expression of approximately 30–70% of human genes [122]. In spite of the aforementioned underrepresentation of microRNA in the content of cancer-related EVs, there is mounting evidence as to their role as biomarkers [123] and biological effectors [57]. As with other macromolecular regulators, the consequences of microRNA sorting into the EV compartment could translate into either cell-autonomous or non-cell-autonomous effects. In the first instance packaging microRNA into EVs effectively relieves the corresponding negative regulation on parental cell transcripts resulting in their rise and, correspondingly, increased functional effects. For example, EV-mediated removal of miR-23b from cancer cells was shown to relieve the molecular breaks on tumor invasion to trigger elevated aggressiveness [57]. 

Once released from cancer cells, microRNA(miRNA)-containing EVs may enter a wide array of recipient cell types and exert biological effects, often of considerable magnitude [104]. Indeed, the aforementioned stochiometric scarcity of EV-associated miRNAs [53] could potentially be compensated for by the catalytic abilities of the miRNAs-containing gene-silencing complexes (RISCs), in which a single miRNA could enter multiple rounds of target interactions [124]. This is consistent with the empirical evidence to suggest that EV-associated miRNAs are capable of negatively regulating a wide range of target genes in EV recipient cells. For example, miR-21, miR-92a and miR-494 act as proangiogenic factors, when transmitted between cells via EVs, in part by relieving the expression of endogenous angiogenesis inhibitors including thrombospondin 1 [125,126,127,128]. In contrast, miR-16 may suppress angiogenesis by downregulating pro-angiogenic VEGF [129]. Similar mechanisms suggested a role of exosomal miRNAs in cancer cell migration, vascular permeability, immunoregulation and metastasis [72,73,130]. In several reports, miRNAs implicated in such effects include miR-181, miR-105, miR-200, miR-203, miR-23 and miR-10b [130,131,132,133,134]. It is noteworthy that EV heterogeneity imparts certain levels of exRNA compartmentalization between different EV subpopulations. For example, Kaur et al. have shown that small non-coding RNA content of CD47^+^ EVs differs from that of CD47^−^ EVs released by the same cells [135]. Thus, subsets of EVs may differ in their content of microRNA and other bioactive RNA biotypes, and similarly diverse would likely be their regulatory functions.

LncRNAs are involved in many important biological processes, such as chromatin modification, gene expression, and nuclear transport, and they may act as regulators of apoptosis, tumor migration, and drug resistance. This is of interest as cancer cells may selectively emit EV-associated chromatin fragments and transfer them to various recipient populations [87,136], a notion that may suggest a parallel emission of lncRNA. Indeed, some lncRNAs are enriched in exosomes, while others are scarcely detectable in the EV cargo, indicating the existence of underlying and poorly defined sorting mechanisms. The gap in the related knowledge is underscored by the fact that while more than 9600 loci in the human genome are classified as putative sources of lncRNAs, less than 100 loci have been characterized for their biological roles [137]. 

Nonetheless, indications as to the role of EV-associated lncRNA in cancer have already begun to emerge. For example, Zhang et al., have shown that lncRNA known as MALAT1 is transferred between cells through an EV-dependent mechanism resulting in the enhancement of cancer cell growth [138]. Similarly, H19 lncRNA was found to be enriched in EVs and stimulated anchorage independent growth of cancer cells upon intercellular transfer [139]. Several other lncRNAs such as lincRNA-p21, HOTAIR, ncRNA-CCND1, TUG1, GAS5 and MALAT1 were also found to be selectively sorted to EVs, often with their levels reflective of DNA damage responses in parental cells [140]. In another study, the telomeric repeat-containing RNA, lncRNA known as TERRA was reported to be packaged into EVs capable of stimulating innate immunity [141]. These are intriguing examples of the roles that may be a part of a larger nexus between the vesiculation pathway and non-cell-autonomous effects of non-coding RNAs.

## 6. EV-Associated Non-Coding RNAs as Biological Mediators in Primary Brain Tumors

Over the past decade the functional and diagnostic significance of EV-associated RNA have also been explored more extensively in primary brain tumors [15,52,96,101,142,143,144]. Much of this effort concentrated on GBM due to the pressing need to improve outcomes in this incurable disease, which is also the most prevalent amongst primary astrocytic brain neoplasms (gliomas) in adults [145]. It is noteworthy that a considerable progress has been achieved in molecular classification of GBM, which is now known to comprise several disease entities, such as proneural, neural, classical and mesenchymal tumors [146] (also recently re-classified as IDH1 mutant tumors and three subgroups of IDH1 wild-type GBMs—RTK I, RTK II and Mesenchymal) [2]. Similarly, the extensive analysis of driver genes involved in GBM progression resulted in identification of several recurrent mutations either common or subtype-specific, including mutant *TERT* promoter, *IDH1* gene, *EGFR* (including *EGFRvIII* deletion mutant), *PDGFRA, PTEN, TP53, CDKN2A/B, ATRX* and several others [2]. While several of these mutations possess a potent transforming potential in experimental systems and some are druggable (e.g., EGFR) this knowledge has yet to translate into more effective therapy. As mentioned earlier, this baffling intractability is likely due to multiple factors, including anatomical challenges of the intracranial disease, the diffuse nature and cellular heterogeneity of GBM, as well as the shortage of reliable biomarkers to track the rapid biological evolution of these devastating tumors [9,147,148] and the complexity of intercellular communications involved in their biology [8,15,16,149]. 

EV-mediated export of non-coding RNA by GBM cells and their stroma attracted considerable interest as both, a part of the intercellular communication network and a source of biomarkers [13]. In this regard EV-mediated intercellular trafficking of microRNA in GBM represents a particularly well studied paradigm [14,15]. GBM cells can act as both donors and recipients of EV-associated regulatory microRNA with the biological net effect resulting from gain- or loss-of-function events imparted by these processes. For example, work of Bronisz and colleagues revealed that with GBM progression glioma stem cells (GSCs) down-regulate the expression of miR-1, which leads to reduced EV-mediated transfer of this microRNA to recipient cells in the tumor microenvironment. Because EV-associated miR-1 appears to control the expression of ANXA2, and a number of other pro-invasive targets (MET) the reduction of its intercellular influence was associated with a non-cell-autonomous increase in GSC invasion, also sphere formation and angiogenic activity [75]. A similar paradigm may apply to a number of tumor suppressive miRNAs deregulated in GBM [14]. On the other hand, GBM cells are enriched in several oncogenic microRNAs including miR-21, miR-23a, miR-30a, miR-221 and miR-451, several of which undergo EV-mediated intercellular trafficking, and may exert tumor promoting effects in recipient cells [14,15,150]. Microglia is among demonstrated targets of GBM derived and EV-associated miRNAs (e.g., miR-21) resulting in modulation of cellular phenotypes [76], but the spectrum of such interactions in this and other brain tumors is very likely much wider [15]. 

MicroRNA networks control several key regulatory programs involved in GBM progression [15]. These effects often regarded as ‘intracellular’ in nature may also be disseminated between cellular populations through miRNA carrying EVs [15,16]. For example, some of the known cellular regulators with oncogenic activity, such as hypoxia-inducible factor 1 (HIF-1), platelet derived growth factor receptor A (PDGFRA) or EGFR, or their effectors, are regulatory targets of miRNAs. Several miRNAs (miR-34a, miR-128, miR-1, miR-26, miR-7) are consistently deregulated in GBM creating permissive conditions for disease progression. EV-mediated intercellular trafficking of the respective miRNAs or their effectors is likely a part of this process at the intercellular level [70,75,151,152].

EV-mediated microRNA export may also regulate the state of the GSC/BITC compartment in GBM. In this case the documented expulsion of miR-1246 and miR-1290 through exosome-like EVs was implicated as one of the mechanisms that maintain cellular stemness, due the role of these miRNAs in neuronal differentiation [153]. EV-mediated control of intracellular and intercellular effects of miRNAs has also been suggested to influence the cellular stemness and malignant progression in pediatric brain tumors [154]. In another study, the loss of miR-128 was shown to be an early event in GBM pathogenesis with several consequences to the GSC molecular landscape [155]. In GBM cells some of the stem cell-related transcription factors such as OCT4 and SOX2 are regulated by miR-148a along with DNA methyltransferase (DNMT) promoter, with a possible impact on the epigenome [156]. Notably high levels of exosomal miR-148a were reported in the serum of GBM patients suggesting at least a possibility that these effects may be transmitted between cellular populations or could be monitored by approaches of liquid biopsy [157]. 

Stromal cells also possess the ability to influence the progression of GBM through the exchange of EV-associated miRNA. For instance, mesenchymal stem cells associated with glioma (GA-MSCs) secrete EVs enriched in miR-1587, which upon entry into cancer cells block nuclear coreceptor (NCOR1) leading to an increase in tumorigenesis [158]. The ability of mesenchymal cell-derived EVs to deliver growth regulatory miRNA to cells (including GBM) inspired a number of studies aiming at mitigating tumor aggressiveness through the use of exosomal carriers of therapeutic RNA (see below) [129,159,160]. 

In contrast to microRNA, the EV-mediated release of other non-coding RNA biotypes has been less studied in brain tumors. This is likely due to poorly understood functional effects of such communication and, at least in some cases, low abundance of some of the ncRNA biotypes in the GBM EV cargo [101]. Nonetheless, several intriguing reports have recently emerged, implicating ncRNA-containing EVs in various aspects of brain tumor biology, such as angiogenesis [161,162]. In this regard, different EV subsets may exhibit considerable variation in the content of ncRNA biotypes and RNA binding proteins. As mentioned earlier, MVs seem to be more reminiscent of parental cells in terms of their repertoire of ribonucleoproteins (RNPs), while exosomes appear to be more distinct [101]. In terms of the relative representation of different RNA biotypes, rRNA and snRNA fragments were reported to be present in more than 1 copy per EV in cultures of GSCs, while mRNA or vRNA are often less abundant (one copy per 10 EVs). This suggests that a massive EV uptake would be required for these less abundant biotypes to exert a robust biological effect [101].

There is, however, an emerging body of evidence as to EV-mediated export and intercellular transfer of glioma-derived lncRNA [101]. Again, several of these reports point to angiogenesis as a regulatory target of glioma-derived EV-associated lncRNA. This trend could be related to either intrinsic properties of lncRNA-containing GBM EVs, or due to the choice of the respective assays and questions. For example, extracellular 5′-tRNA fragments (5′-tRFs, 30–32 nt-long), also called 5′-tRNA halves (tiRNAs), are produced in glioma cells by ribonuclease known as angiogenin (ANG) [161]. Interestingly, the enrichment of both ANG and 5′-tRFs is observed in GSC-derived exosomes suggesting that tRNA cleavage may occur within these EVs and outside of parental cells [101]. It is noteworthy that ANG was discovered as a stimulator of vascular growth [163,164], but whether this is related to the EV-associated ribonuclease activity is presently unclear. It is thought provoking, however, that another lncRNA known as POU class 3 homeobox 3 (POU3F3) overexpressed in glioma cells is also sorted into exosome-like EVs and plays a documented role in angiogenic responses [162]. Similarly, the extracellular export was documented for the long intergenic non-coding RNA (lincRNA) known as CCAT2 (linc-CCAT2) and frequently upregulated in glioma cells. This export involves tumor-derived EVs and their transfer to endothelial cells, where linc-CCAT2 is implicated in stimulation of cell survival and angiogenic responses [165]. In another study, lncRNA known as HOTAIR, which possesses oncogenic activity, was found to activate at least two pro-angiogenic mechanisms, namely through the induction of VEGF-A in glioma cells themselves, and by a direct, EV-mediated trafficking to endothelial cells [166]. These and other examples illustrate the emerging role of extracellular RNA biotypes, especially non-coding RNA species, in intercellular communication and regulation of processes of central importance in the pathogenesis of primary brain tumors.

## 7. EV-Associated Non-Coding RNA in Metastatic Brain Tumors

Metastatic brain tumors represent a prevalent and daunting clinical challenge and are, in many respects, a biological enigma [4]. This is because several factors conspire to recruit, retain and sustain ectopic cancer cells in the unique intracranial microenvironment shielded by the blood–brain barrier (BBB) and unique immune and growth regulatory influences [167,168,169]. EVs emerged as a mechanism involved in breaching some of these defenses through induction of vascular permeability, triggering neuroinflammation and formation of a supportive cancer cell niche [35,71,72,170]. Among several unique EV properties that enable them to play such roles, it is worth mentioning their ability to traverse the BBB [171], modulate or damage endothelial cells [170], trigger prothrombotic states within the vasculature [172] and mediate growth-promoting and immunosuppressive intercellular interactions [73,77]. 

Indeed, at least some of these effects involve the trafficking of EV-associated non-coding RNA. For example, in breast cancer models the release of EV-associated miR-181c and its entry into endothelial cells downregulates 3-phosphoinositide dependent protein kinase 1 (PDPK1) resulting in disruption of cofilin regulated actin dynamics and local inactivation of BBB. This process paves the way to brain metastasis [72]. Similarly, breast cancer cell derived EVs that carry miR-105 downregulate ZO-1 protein, thereby obliterating tight junctions. This change disorganizes sprouting processes of the endothelium and facilitates cancer cell metastasis to the brain and other organs [130]. Another mechanism, by which breast cancer cells may control metastatic progression, is by the release of EV-associated miR-122, which reprograms the metabolism of distant tissues, such as brain and lung and promotes cancer cell dissemination to the respective organ sites [173]. Conversely, astrocyte-derived EVs carrying elements of the miR-17-92 cluster were recently reported to penetrate into silent breast cancer cells lodged in the brain parenchyma. Remarkably, the resulting downregulation of PTEN expression triggered overt metastatic growth of these cells. This effect could be recapitulated by a direct delivery of miR-19a into cancer cells [73], suggesting that PTEN loss may possess a more general significance for tumorigenesis in the brain [82]. Whether other cancers frequently metastasizing to the brain (lung, melanoma) employ these or other mechanisms remains to be elucidated.

## 8. EV-Associated Non-Coding RNA as Emerging Biomarker Platform in Brain Malignancies

One of the unique opportunities associated with the process of EV release from cancer cells and their stroma lies in the domain of liquid biopsy [174]. This is because EVs contain combinatorial assemblies of molecular identifiers of multiple salient features associated with the malignant process, such as cellular identity, state, stress, oncogenic drivers or markers of drug resistance, all of which could be exploited as a natural multiplexing mechanism in brain tumor diagnosis [16]. For example, co-expression of a specific ncRNA and stem cell markers in circulating EVs may inform about the state of this RNA in the cancer stem cell population in that tumor, samples of which may not be surgically inaccessible. The advantages of this approach also include the constant EV shedding into the biofluid spaces such as blood or cerebrospinal fluid, their numerical abundance, relative stability of the EV cargo due protective effects of the lipid membrane [144], and their role as portals for the uptake of cancer-related molecular cargo by the potentially usable secondary reservoirs of this information, such as platelets [86] or leukocytes [87]. Indeed, several studies have already demonstrated the accessibility of EV-associated molecular information in samples of cerebrospinal fluid and blood of experimental animals and brain tumor patients [52,68,144]. This information could potentially reveal, in real time and in a non-invasive manner, several crucial characteristics of the evolving malignancy, such as the molecular subtype, emerging drug targets or therapeutic resistance, immunological status, angiogenic profile, stem cell signals and many other characteristics [9,33,77,81,114,143,175]. 

The aforementioned combinatorial co-expression patterns of diagnostically informative macromolecules on the surface or in in the cargo of brain tumor EVs also presents some additional and unique advantages. For example, this property fundamentally separates EVs from soluble analytes detectable in plasma (e.g., RNPs), which often cannot be unambiguously assigned to their cellular sources, or simply represent effects of cellular and molecular breakdown. While circulating cancer cells possess the required complexity and are sometimes detectable in blood of patients with GBM [176] or medulloblastoma [177], they are exceedingly rare (less than 10 per 1 mL of blood) and thereby may not represent the true extent of cellular heterogeneity of the underlying disease [174]. In contrast, tumor EVs may reach 10^12^ per mL of blood of cancer patients and collect information from all perfused primary and metastatic sites [13,178]. 

It should be noted, however, that while EVs may have considerable advantages as a biomarker platform, they also pose multiple challenges. Among those of special consideration is the ‘noise’ generated by the admixture of endogenous EVs in biofluid preparations, EV heterogeneity, the extent of which is presently unknown, short half-life of EVs in the circulation, and other factors that may collectively impact the amplitude and reproducibility of EV-associated diagnostic signals [175]. While in brain tumors multiple classes of EV-associated molecules may possess a diagnostic value (lipids, metabolites, cytokines, integral proteins and oncoproteins, DNA and RNA) [142,179], both coding and non-coding RNAs have recently attracted considerable investigative attention and deserve more discussion [96]. 

EV-associated ncRNAs could provide a unique window into the status of cancer cells due to their greater abundance than that of mRNA (even 20-fold higher levels in some cases) in the EV cargo [101] and the expression patterns that may accurately reflect cellular phenotypes [15]. Importantly, certain ncRNAs biotypes, such as lincRNA, are expressed in a tissue-specific manner relative to lesser specificity of mRNAs [180]. Subsets of ncRNA may also be enriched in the EV cargo in ways that may be informative as to the state of parental cells, even if these patterns are often not identical (between cells and their derived EVs) [150]. Thus, algorithms to extract clinically relevant information from brain tumor-derived EVs isolated from biofluids represent a point of considerable interest and research efforts [175]. 

Several studies suggest the feasibility of the EV-based analysis of ncRNA for the purpose of biomarker development in brain tumors (Table 1). For example, miR-21 is often viewed as oncogenic in the context of GBM due to its ability to down regulate various tumor suppressor genes, including PTEN, metalloproteinase inhibitor 3 (TIMP3), reversion-inducing cysteine-rich protein with Kazal motifs (RECK) [45], and programmed cell death protein 4 (PDCD4) among others [15,181,182,183]. Consequently, several studies evaluated the upregulation of vesicular miR-21 in serum [52] and cerebrospinal fluid [184,185] of GBM patients and demonstrated appreciable sensitivity and specificity of this assay. In this case cerebrospinal fluid rather than serum appears to be a preferable source of the EV-associated miR-21 signal, likely due to cancer cell proximity. Similarly, downregulation of miR-1 during GBM progression is reflected by the levels of this miRNA in EVs [75], along with profiles of other miRNAs [175]. In a series of studies several miRNAs, such as miR-21, miR-222, miR-124-3p, miR-221, miR-320, miR-574-3p, and miR-301a were found to be upregulated in EVs derived from serum of high-grade glioma patients [186,187,188,189]. 

In some cases, EV-associated ncRNAs may potentially facilitate differential diagnosis. For example, vesicular mir-301a was only upregulated in the context of GBM and not in other brain tumors, such as meningioma, primary central nervous system lymphoma, or pituitary adenoma [188]. In another study the combined upregulation of miR-10b and miR-21 in the cerebrospinal fluid was found in patients with either GBM, or brain metastases, while members of the miR-200 family were present only in patients with metastatic brain cancer [192]. Similarly, EV-associated miR-151 in the cerebrospinal fluid of GBM patients correlated with a poor response to temozolomide (TMZ) therapy suggesting that such tests may separate therapeutic responders and non-responders [9,193].

While the aforementioned reports highlight mostly the diagnostic potential of miRNA in primary and metastatic brain tumors, other vesicular ncRNA biotypes have only scarcely been studied. This is an urgent priority given the preponderance of other than miRNA biotypes of ncRNA in the EV cargo [101]. The feasibility of such approaches has been explored through studies on the EV-mediated export of some of the plausible ncRNA known to be upregulated in cultured brain tumors cells, such as POU3F3 or CCAT2 [162,165]. In addition, snRNA known as RNU6-1 was detected in serum EVs in glioblastoma patients and distinguished them from healthy controls [189]. As mentioned earlier HOTAIR was found to be elevated in EVs, but not in the soluble fraction of serum of GBM patients [191]. HOTAIR signal disappeared from blood after surgical tumor resection and re-emerged at GBM recurrence suggesting that EV mediated release of this ncRNA is reflective of the tumor burden [191]. These examples illustrate the role of vesiculation pathways in formation of the ncRNA patterns in biofluids and highlights the related opportunities to develop liquid biopsy approaches to diagnose and monitor brain tumor progression. 

## 9. EVs as Carriers of Therapeutic Non-Coding RNA

The ability of endogenous ncRNA to be delivered to various cellular targets through EV-mediated release-uptake mechanisms [194] inspired considerable efforts to exploit these processes for therapy [171]. Indeed, various ways of loading exogenous molecular cargo to exosomes for the purpose of delivering therapeutic payloads to target cells have been explored [195], revealing both promise [171] and challenges of this paradigm. [196]. 

The majority of studies involving EV-based RNA therapy focused on gene-silencing approaches including those predicated on small interfering RNAs (siRNAs), shRNA (short hairpin RNA) and miRNA [197,198]. For example, pioneering studies by Alvarez-Erviti and colleagues documented the feasibility of engineering EV donor cells to express Lamp2b-RVG fusion product directing such EVs into the brain, where they delivered pre-loaded functional siRNA [171]. Likewise, targeting mutant driver genes in cancer, such as MYC and KRAS led to encouraging preclinical results [199,200]. In some of these studies exogenous siRNA was introduced into EVs using electroporation [171,201], but efforts are underway to improve on this technique and make the process more efficient [202]. One such alternative exploits the endogenous cellular machinery for sorting RNA cargo into EVs. This strategy involves transfection of a shRNA expression vectors or synthetic siRNA into parental cells, after which siRNA-carrying EVs can be directly isolated from cell culture supernatants [203]. Another central question in this emerging technology is the selection of target genes, of which several have been explored experimentally, including the aforementioned oncogenes as well as hepatitis C virus, protein kinase-1, RAD 51, RAD 52 and others [201,204,205]. In this regard one of the challenges associated with the EV-mediated gene-silencing approach is the stoichiometry of different targeting RNA ‘warheads’ to their specific cellular transcripts. It is suggested that ∼3000 siRNAs per EV would be required for optimal effects. However, overloading may induce aggregation, compromise vesicle integrity, and impede productive gene silencing [206,207,208,209]. Using other silencing reagents, such as microRNA could overcome some of these limitations due to catalytic properties of RISC, as discussed earlier [124]. 

One research area where the interest in EV-mediated delivery of ncRNA has been especially intense is the use of mesenchymal stem cell (MSCs) as biological modifiers. It is believed that if EVs were able to recapitulate some of the homeostatic effects of their donor cells they could represent an advantageous alternative to cell therapy due to the favorable immunological, biosafety and handling considerations. Examples of studies that pursued these concepts include delivery of MSC derived exosomes containing tumor suppressor, miR-146b, into GBM xenografts resulting in anti-proliferative effects [160]. Similarly, MSC-derived exosomes containing anti-miRs against oncogenic miR-9 increased drug sensitivity of GBM when treated in combination with the chemotherapeutic agent, temozolomide [159]. 

While these opportunities are still relatively unexplored in the context of brain tumors, in other areas the therapeutic potential of EV-associated ncRNA is being vigorously pursued. For example, in a recent study MSC-derived EVs enriched for miR-21a-5p were transferred into the myocardium and shown to exert a cardioprotective effect dependent on targeting multiple pathways [209]. Encapsulation of miR-124 in EV shuttled to hepatic stellate cells resulted in the reduced expression of *CCN2*, a gene known to be associated with liver fibrosis [207]. Similarly, miR-122 delivered to hepatocellular carcinoma cells via EVs resulted in the inhibition of tumor growth and increased drug sensitivity [208]. These reports along with many others signal the promise of EVs as vehicles of RNA-based therapeutics. 

## 10. Concluding Remarks

In this article we surveyed some of the emerging evidence linking pathways of cellular vesiculation and ncRNA exit from brain cancer and discussed trafficking of this material between cells and in biofluids. These processes are of potential significance in the biology and diagnosis of brain malignancies where driver mutations, changes in the epigenome and in the microenvironment fuel the disease process, while profoundly affecting ncRNA networks. We highlighted the fact that while a significant proportion of the related literature explores these processes in GBM, other classes of primary brain tumors, such lower grade gliomas, medulloblastoma, ependymoma, embryonal brain tumors and tumors derived from lymphoid, vascular or metastatic cells likely operate as EV communication networks as well, with EV-associated ncRNA as a part of the effector machinery and intercellular interactome [210,211]. 

Brain tumor-derived EVs represent a unique repository of ncRNAs reflective of cellular state and transformation status. Passage of this material through biofluids, such as blood and cerebrospinal fluid, as well as its retention within the accessible cellular reservoirs (platelets, leukocytes [86,87]) represents, as we argued, a unique opportunity to sample and diagnostically interpret these ncRNA profiles. In this regard, EVs may offer several advantages in comparison to other liquid biopsy platforms. The related efforts, initiated more than a decade ago [50,51,52], are well underway with multiple groups developing new technologies, some close to clinical translation, through investigator-driven programs [143], consortia (https://commonfund.nih.gov/exrna) and private sector interests [212]. There is also a considerable interest in using EVs and their encapsulated natural or artificial ncRNA cargo as anticancer therapeutics [200].

While there are many reasons for optimism in this emerging field, challenges do exist, as do risks associated with manipulation of highly complex and heterogeneous nano-objects, such as EVs. In this regard, the adherence to analytical standards of reproducibility and community guidelines is of paramount importance to ensure the generation of robust and widely applicable data, including EV analysis and ncRNA profiling [213,214,215]. Importantly, a sober assessment of opportunities, pitfalls, relative roles of EVs, as well as their functional and diagnostic context-specific limitations in brain tumors is essential for a long-term success of this fascinating and promising area of investigation.

## Figures and Tables

**Table 1:** List of selected EV-associated ncRNA biomarkers under investigation in brain tumors.

Name	Source	Detection Method	Reference
**miRNAs**			
miR-1290, miR-1246	Pediatric glioma stem cells	Microarray and qRT-PCR	[154]
miR-21	Serum	qRT-PCR	[52]
miR-21	Cerebrospinal fluid	qRT-PCR	[184,185]
miR-21, miR-222, miR-124-3p	Serum (World Health Organization (WHO) Grade I–IV GBM, post-surgical resection)	qRT-PCR	[186]
miR-210	Metastatic (brain-tropic 70W, MDA-MB-231BR, and CTC1BMSM variants), Non-metastatic (non-BM MeWo, MDA-MB-231P and CTC1P)	MicroRNA PCR array	[190]
miR-221	Serum (WHO Grade I–IV GBM) and cells (SHG-44, U251, U87MG)	qRT-PCR	[187]
miR-301a	Serum (WHO Grade I–IV GBM, post-surgical resection, recurrence)	qRT-PCR	[188]
miR-320, miR-574-3p	Serum	qRT-PCR	[189]
**Other ncRNAs**			
HOTAIR	Serum	qRT-PCR	[191]
linc-CCAT2	Cells (U87MG)	qRT-PCR	[165]
linc-POU3F3	Cells (A172)	qRT-PCR	[162]
RNU6-1	Serum	qRT-PCR	[189]
